# Fast discharge process of layered cobalt oxides due to high Na^+^ diffusion

**DOI:** 10.1038/srep09006

**Published:** 2015-03-11

**Authors:** Takayuki Shibata, Yuya Fukuzumi, Wataru Kobayashi, Yutaka Moritomo

**Affiliations:** 1 Fucalty of Pure and Applied Science, Univ. of Tsukuba, Tsukuba 305-8571, Japan; 2 College of Physics, Univ. of Tsukuba, Tsukuba 305-8571, Japan; 3 Center for Integrated Research in Fundamental Science and Engineering (CiRfSE), Univ. of Tsukuba, Tsukuba 305-8571, Japan

## Abstract

Sodium ion secondary battery (SIB) is a low-cost and ubiquitous secondary battery for next-generation large-scale energy storage. The diffusion process of large Na^+^ (ionic radius is 1.12 Å), however, is considered to be slower than that of small Li^+^ (0.76 Å). This would be a serious disadvantage of SIB as compared with the Lithium ion secondary battery (LIB). By means of the electrochemical impedance spectroscopy (EIS), we determined the diffusion constant (*D*) of Na^+^ in thin films of O3- and P2-type NaCoO_2 _with layered structures. We found that the *D* values (~ 0.5–1.5 × 10^−10^ cm^2^/s) of Na^+ ^are higher than those (< 1 × 10^−11^ cm^2^/s) of Li^+^ in layered LiCoO_2_. Especially, the *D* values of O3-NaCoO_2 _are even higher than those of P2-NaCoO_2,_ probably because O3-NaCoO_2 _shows successive structural phase transitions from the O3, O’3, P’3, to P3 phases with Na^+^ deintercalation. We further found that the activation energy (*E_D_* ~ 0.4 eV) for the Na^+^ diffusion is significantly low in these layered cobalt oxides. We found a close relation between the relative capacity and the renormalized discharge rate ( = *L*^2^/*DT*, where *L* and *T* are the film thickness and discharge time, respectively).

The SIBs store electric energy by the intercalation/deintercalation of abundant Na^+^ (Clark number = 2.63), and hence, are suitable for large-scale batteries that will enable the stable use of solar and wind energies^1^,^2^,^3^. Recently, Komaba et al.^4^ have found that hard carbon shows a high capacity of more than 200 mAh/g and good cyclability. This finding opens the door to the commercial utilization of SIB technologies, and hence, significantly stimulates investigation and exploration of cathode materials for SIBs. Among the cathode materials for SIBs^1^,^2^, layered transition metal oxides (Na*M*O_2_, where *M* is transition metal) show promising electrochemical properties^5^,^6^ as well as rich structural properties^7^,^8^,^9^. We have much wider selections of sodium compounds, *e.g*., transition metal and stacking structure, as compared with those of the lithium compounds. For example, O3-NaCrO_2_ shows a capacity of 120 mAh/g and an average operating voltage of 3.0 V (vs. Na) with good cyclability in SIBs^10^ while isostructural O3-LiCrO_2_ shows a poor capacity and cyclability in LIBs. In addition to the O3-type staking, *i.e.*, close-packed AB|CA|BC stacking of oxygen sheets along the *c*-axis, the sodium compounds frequently exhibit the P2-type staking, *i.e*., close-packed AB|BA stacking of oxygen sheets. For example, P2-Na_0.6_MnO_2_^11^ shows a capacity of 140 mAh/g and an average operating voltage of 2.5 V (vs. Na). The Na deficiency of the P2-type compound, however, is serious drawback in the actual process of manufacture, because an extra electrochemical process is needed to compensate the Na deficiency.

SIBs are considered for stationaly energy storage bacause the diffusion process of large Na^+^ (ionic radius is 1.12 Å) is believed to be slower than that of small Li^+^ (0.76 Å). However, the Na^+^ diffusion is really dependent on the crystal structure. For example, the Na^+^ diffusion in P2-Na_0.66_Li_0.22_Ti_0.78_O_2_ with layered structure^12^ and Na_3_V_2_(PO_4_)_3_^13^ with NASICON (Na Super Ionic CONductor) structure are considerably fast, while it is much slower in Li_4_Ti_5_O_12_^14^,^15^ with spinel structure. In order to precisely determine the Na^+^ diffusion constant (*D*), we fabricated thin films of O3- and P2-type NaCoO_2 _on an Au collector electrode by means of the pulsed laser deposition (PLD) method. We found that the *D* values (~ 0.5–1.5 × 10^−10^ cm^2^/s) of Na^+ ^are higher than those (1 × 10^−11^ cm^2^/s^16^) of Li^+^ in layered LiCoO_2_. We further extracted a close relation between the relative capacity and the renormalized discharge rate ( = *L*^2^/*DT*), with eliminating the voltage drop effects, *i.e.*, -*IR*, in the rate-dependence of discharge curves.

We first determined the crystal structures of O3-type Na_0.99_CoO_2_ and P2-type Na_0.67_CoO_2_ by Rietveld analysis (Rietan-FP^17^) based on the synchrotron-radiation X-ray powder diffraction pattern obtained at BL02B2 beamline^18^ of SPring-8, Japan (Figs. S1 and S2). Figure 1 shows the Na^+^ diffusion path of (a) O3-type Na_0.99_CoO_2_ and (b) P2-type Na_0.67_CoO_2 _together with the actual inter-oxygen distances. In the O3-type stacking, Na^+^ is located in the oxygen octahedron and migrates to the neighboring site through the oxygen isosceles triangle, 2.84 Å in base and 3.64 Å in oblique side^19^. The triangle window is rather narrow for Na^+^: the optimal distance ( = 1.97 Å) to the three vertexes is much smaller than the sum ( = 2.52 Å) of the ionic radii of Na^+^ (1.12 Å) and O^2−^ (1.4 Å). In the P2-type stacking, Na^+^ is located in oxygen triangular prism and migrates to the neighboring site through the oxygen rectangle, 2.83 Å in base and 3.57 Å in height. The rectangle window is wide for Na^+^: the optimal length ( = 2.28 Å) to the four vertexes is comparable the sum ( = 2.52 Å) of the ionic radii. Thus, the Na^+^ diffusion is considered to be faster in the P2-type stacking with oxygen prisms.

In order to determine *D* and ionic charge-transfer resistance (*R*_ct_), we performed the electrochemical impedance spectroscopy (EIS) of thin films of O3-NaCoO_2 _and P2-NaCoO_2 _(Figs. S5 and S6). The EIS curves were analyzed by least-squares fitting with the Randle equivalent circuit [Fig. 2(a)]. In the analysis, we used a constant phase element (CPE)-restricted diffusion impedance, 
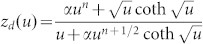
 and 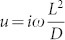
20. As shown in Fig.2 (b), this model well reproduced the overall feature of the EIS curve, *i.e.,* semicircle in the high-frequency region and bending behavior in the low frequency region. Especially, the CPE-restricted diffusion impedance reproduced the finite slope in the low frequency limit. Thus obtained *D* and *R*_ct_ were plotted in Figs. 2(c) and (d). The *D* values of O3-NaCoO_2_ are even higher than those of P2-NaCoO_2 _[Fig. 2(c)], the origin of which will be discussed later. Importantly, the *D* values (~ 0.5–1.5 × 10^−10^ cm^2^/s) of Na^+^ in layered cobalt oxides are much higher than those (< 1 × 10^−11^ cm^2^/s^16^) of Li^+^ in LiCoO_2_. We note that the experimentally-obtained *D* values in layered cobalt oxides are comparable to the calculated one (~ 2.1 × 10^−10^ cm^2^/s^12^) of P2-Na_0.66_Li_0.22_Ti_0.78_O_2_. In the high-*x* region (*x* > 0.82) of P2-Na*_x_*CoO_2_, the high-frequency semicircle is too deformed to be analyzed at 303 K (Fig. S5). The EIS curves of P2-Na*_x_*CoO_2 _(*x* = 0.5) show no bending behavior in the low frequency region in the temperature range from 303 K to 349 K (Fig. S6). This implies that the *D* value at *x* = 0.5 is much higher than those at *x* = 0.54 − 0.72. This is probably due to the Na^+^/vacancy ordering. On the other hand, the *R*_ct _values of O3-NaCoO_2_ are much smaller than those of P2-NaCoO_2_.We further estimated the activation energies of *D* and *R*_ct_ by Arrhenius plot (Fig. S7). In the high-*x* region (*x* > 0.82) of P2-Na_x_CoO_2_, the activation energies of *D* and *R*_ct_ can be evaluated because the deformations of the EIS curves are released above 318 K (Fig. S6). The activation energies (*E*_D _~ 0.4 eV) of *D* in O3-NaCoO_2_ are comparable to those in P2-NaCoO_2_. On the other hand, the activation energies (*E*_R _~ 0.3 eV) of *R*_ct_ in O3-NaCoO_2_ are slightly lower than those (~ 0.4 eV) in P2-NaCoO_2_.

The open-current-voltage (OCV) discharge curve slightly different between O3-type and P2-type NaCoO_2_ (black curves in Fig.3). The layered cobalt oxides are known to show successive structural phase transition with Na^+^ intercalation/deintercalation. The phase transition causes the voltage drop at single phase point and the voltage plateau in two phase region. Lie. *et al.*^21^ systematically investigated the structural properties of O3-Na*_x_*CoO_2_ against *x*. They found four phases, *i.e.,* the O3 (*x* = 1.00), O’3 (monoclinic phase: *x* = 0.83), P’3 (monoclinic phase: *x* = 0.67), and P3 (*x* ~0.5) phases. In the O’3phase, Na^+^ is located in the oxygen octahedron as in the O3-type. In the P’3 and P3 phases, Na^+^ is located in the oxygen prisms as in the P2-type. Actually, we observed four voltage drops in the discharge curve of O3-NaCoO_2_, which correspond to the O3, O’3, P’3 and P3 phases (Fig. S8). In addition, the inter CoO_2_-sheet distance (~ 5.44 Å) of the oxidized film (*x* = 0.65) is much longer than that (~ 5.14 Å) of the as-grown film, (Fig. S3) suggesting the P3-type structure^21^. These observations indicate that the O3-Na*_x_*CoO_2_ film (0.5 < *x* < 0.83) contains the P3 (or P’3) phases. Then, the unexpected high-*D* values observed in O3-NaCoO_2 _[see Fig.3(c)] are ascribed to the P3-type host framework with oxygen prisms. On the other hand, Berthelot *et al.*^8^ investigated the structural properties of P2-Na*_x_*CoO_2_ against *x*. They found three phases, *i.e.,* the *x* = 0.72, 0.76, and 0.79 phases, in addition to the well-known *x* = 1/2 and 2/3 phases. These phases are ascribed to the Na^+^/vacancy ordering within the P2-type host framework. We observed three voltage drops in the discharge curve of P2-NaCoO_2_, which correspond to the 1/2, 3/2, and ~ 0.76 phases (Fig. S8).

Figure 3 shows discharge curve of (a) O3-NaCoO_2_ and (b) P2-NaCoO_2_ at various discharge rates. The fast discharge properties observed in thin films are probably ascribed to the ideal electronic contact between the active material and the collector electrode. Generally, the discharge process is governed by the electric conductivity (or voltage drop) and Na^+^ diffusion process. We first eliminated the voltage drop effect (-*IR*: *I* and *R* is the current density and battery resistances) by changing the cut-off voltage for evaluation of the capacity (*Q*) as 2.5 V - *IR*. The *R* values are 477 Ω in Ο3-NaCoO_2 _(*L* = 320 nm), 660 Ω in Ο3-NaCoO_2 _(*L* = 330 nm), and 1160 Ω in Ο3-NaCoO_2 _(*L* = 170 nm). Then, the residual rate-dependence can be ascribed to the Na^+^ diffusion effect. Here, let us consider one-dimensional diffusion equation,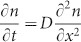, where *n* is the Na^+^ density. We introduce dimensionless time and depth scales, *i.e.*, α ( = *x*/*L*) and β ( = *t*/*T*), where *T* is the time needed for the full discharge. In other words, system is fully-discharged at β = 1. Then, we obtained a renormalized diffusion equation, 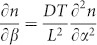. This equation indicates that the diffusion dynamics scales with the renormalized discharge rate (*L*^2^/*DT*), irrespective of the individual *L*, *D*, and *T* values.

Figure 4 shows relative capacities (*Q*/*Q*_0_: *Q*_0_ is the OCV capacity) of three films against *L*^2^/*DT*. The *Q*/*Q*_0_ value begins to decrease when *L*^2^/*DT* approaches ~ 0.1 while it remain nearly ~ 1 in the small-*L*^2^/*DT* region. The decrease, commonly observed in three films, should be ascribed to the Na^+^ diffusion process. The solid curve in Fig.4 is the result of simulation of discharge process. The simulation was performed by difference calculus with the space mesh (*N_α_*) of 30 and time mesh (*N_β_*) of 20,000 (Fig. S9). At the active material/electrolyte boundary (*α* = 0), we forces a constant Na^+^ intercalation ( = *N_α_* /*N_β_*). The Na^+^ intercalation stops if the density ( = 1 – *n*) of the Na^+^ vacancy at *α* = 0 becomes smaller than *N_α_* /*N_β_*. The *β* value at this condition corresponds to *Q*/*Q*_0_. The simulated curve begins to decreases at *L*^2^/*DT* ~ 0.1, and well reproduces the global feature of the experiment.

In summary, we fabricated thin films of O3- and P2-type NaCoO_2_ by means of the PLD method and precisely determined the magnitudes and activation energies of *D* and *R*_CT_. We found that the *D* values (~ 0.5–1.5 × 10^−10^ cm^2^/s) for the Na^+^ diffusion are much higher than those (< 1×10^−11^ cm^2^/s^16^) for the Li^+^ diffusion in layered cobalt oxides. Especially, the *D* values of O3-NaCoO_2 _are even higher than those of P2-NaCoO_2. _This is probably because oxidized O3-Na*_x_*CoO_2 _film (0.5 < *x* < 0.83) contains the P3-type host framework with oxygen prisms. We further found that the activation energy (*E_D_*~ 0.4 eV) for the Na^+^ diffusion is significantly low in these layered cobalt oxides. Our experiment indicates that we can explore active materials with high Na^+^ diffusion even though the diffusion process of larger Na^+^ was believed to slower than that of small Li^+^.

## Method

### Film synthesis and characterization

This film of O3-NaCoO_2_ (P2-NaCoO_2_) was grown on Au-deposited MgO (100) substrate at 823 K (923 K) in an oxygen partial pressure of 0.01 Pa (50 Pa) for 10–30 min by pulsed laser deposition (PLD) method. The film thickness (*L*) was 200–400 nm, and the film area was 0.5 cm^2^. *L* was determined by cross-sectional SEM image, after the electrochemical measurements. The mass of the active material was estimated by *L* and the actual density of the film, which was evaluated with a larger and thicker film. The second harmonics of an yttrium–aluminum–garnet (YAG) pulse laser was used as excitation light source. The pulse energy, repetition frequency, and wavelength were 2.0 J/cm^2^, 10 Hz, and 532 nm, respectively. The distance between the target and the substrate was 35 mm. The sodium concentration of the film was assumed to be the same as the target, which was determined by Rietveld structural analysis (Figs. S1 and S2). The targets of O3-Na_0.99_CoO_2_ (P2-Na_0.67_CoO_2_) were prepared by solid state reaction. First, Na_2_O_2_ (Na_2_CO_3_) and Co_3_O_4_ were mixed in a 1.25: 1.0 (0.7: 1.0) atomic ratio and calcined at 823 K (1073 K) for 16 h (12 h) in O_2_ atmosphere (air). Then, the O3-NaCoO_2_ (P2-NaCoO_2_) powder was finely ground, pressed into pellets with 2 mm in diameter, and calcined at 823 K (1073 K) for 16 h (12 h) in O_2_ atmosphere (air). We confirmed the (003) and (006) [(002) and (004)] reflections in the X-ray diffraction pattern (Fig. S3) of O3-NaCoO_2_ (P2-NaCoO_2_), indicating the (001)-orientation of the films. The scanning electron microscope (Fig. S4) revealed that the O3-NaCoO_2_ (P2-NaCoO_2_) film consists of grains of ~ 100 nm (300–500 nm) in diameter.

### Electrochemical measurement

The discharge properties were investigated using a two-pole beaker type cell with a battery charge/discharge system (HOKUTO HJ-SD8). The cathode and anode were the film and Na, respectively. The electrolyte was propylene carbonate (PC) containing 1 mol/L NaClO_4_.The lower and upper cut-off voltages vs. Na were 1.5 V (2.0 V) and 3.8 V (3.4 V) for O3-NaCoO_2_ (P2-NaCoO_2_), respectively. The charge current was set to 4 μA/cm^2^. The electrochemical impedance spectroscopy (EIS) was carried out with a potentiostat (BioLogic VSP). The frequency range was from 5 mHz to 200 kHz, and the amplitude was 30 mV.

### Analysis of the EIS data

**Figure 2:**
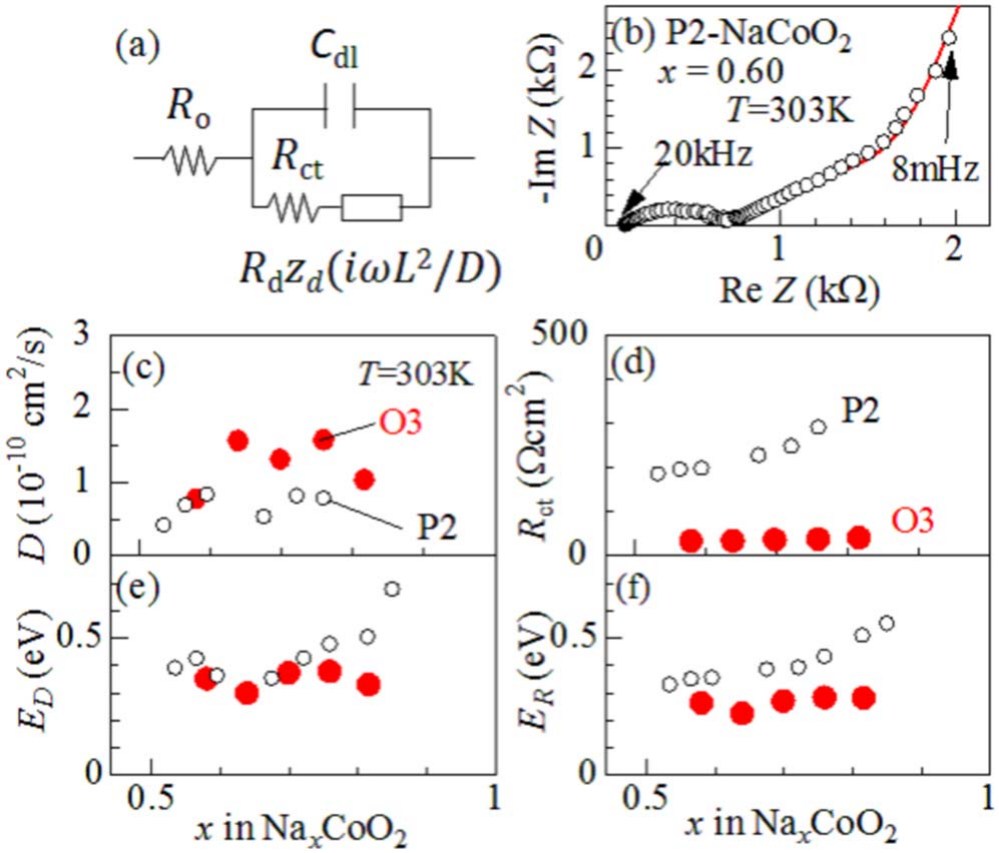
Na^+^ diffusion constant (*D*) and ionic charge-transfer resistance (*R*_ct_) of layered cobalt oxides. (a) Randles equivalent circuit: high frequency resistance of electrolyte (*R*_o_), ionic charge-transfer resistance (*R*_ct_), double layer capacitance (*C*_dl_), and restricted diffusion. impedance [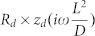, *R*_d_ and *z*_d _are characteristic resistance and reduced diffusion impedance, respectively] of flat plate with thickness *L*. (b) Complex impedance of P2-NaCoO_2_ at *x* = 0.60. Red curve is results of the least-squares fitting of with the Randles equivalent circuit: *R*_o_ = 170 Ω, *R*_ct_ = 460 Ω, *C*_dl_ = 0.62 μF, *R*_d_ = 2500 Ω, *D* = 0.83 × 10^−10^ cm^2^/s, α = 0.40 and *n* = 0.77. (c) *D*, (d) *R*_ct_ (e) activation energy (*E_D_*) of *D*, and (f) activation energy (*E_R_*) of *R*_ct_. against *x*.

The EIS data were analyzed by Randles equivalent circuit [see Fig. 2(a)] with high frequency resistance of electrolyte (*R*_o_), ionic charge-transfer resistance (*R*_ct_), double layer capacitance (*C*_dl_), and restricted diffusion impedance [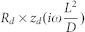, *R*_d_ and *z*_d _are characteristic resistance and reduced diffusion impedance, respectively] of flat plate with thickness *L*. We used the CPE-restricted form, 
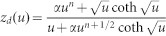
 and 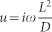
20. The CPE-restricted diffusion impedance becomes the well-known forms in special cases: 
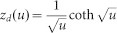
 at 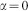 (perfectly-reflective boundary) and 
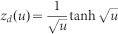
 at 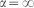
(perfectly-absorptive boundary). The seven parameters, *i.e*., *R*_o_, *R*_ct_, *C*_dl_, *R*_D_, *α*, *n*, and *D*, were determined by least-squares fittings of the EIS curves.

## Author Contributions

Y. M. planed the overall the experiment and wrote the main manuscript. T. S. synthesized the thin films and analyzed the impedance spectra. K. W. fabricated the PLD chamber and Y. F. make a least-squares fitting program with the CPE-restricted diffusion impedance.

## Supplementary Material

Supplementary Information Supplementary Information

## Figures and Tables

**Figure 1:**
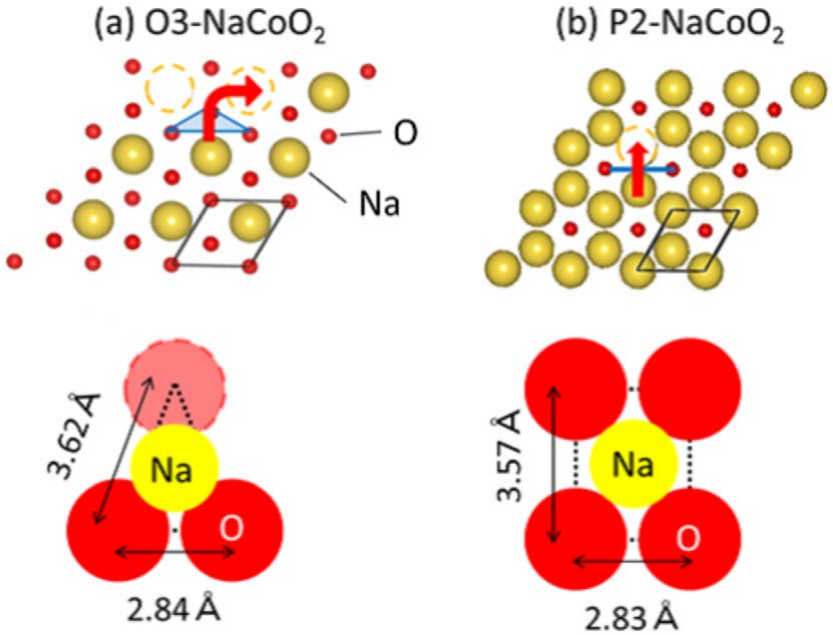
Na^+^ diffusion path of (a) O3-type Na_0.99_CoO_2_ and (b) P2-type Na_0.67_CoO_2_. Upper panels show Na^+^ layers (large yellow spheres) together with the sandwiching oxygen layers (small red spheres). Red arrows and blue marks indicate the Na^+^ diffusion path and the oxygen window, respectively. Lower panels show the oxygen window together with the ionic radii of Na^+^ (1.12 Å) and O^2−^ (1.40 Å).

**Figure 3:**
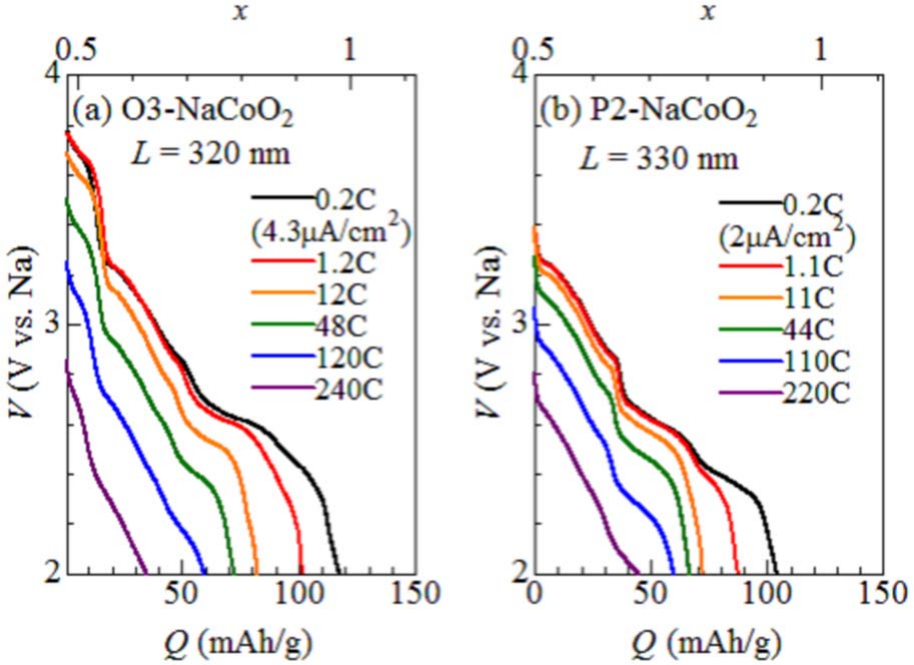
Discharge curves of films of (a) O3-NaCoO_2_ and (b) P2-NaCoO_2_. Film thicknesses (*L*) are 320 nm and 330 nm, respectively.

**Figure 4:**
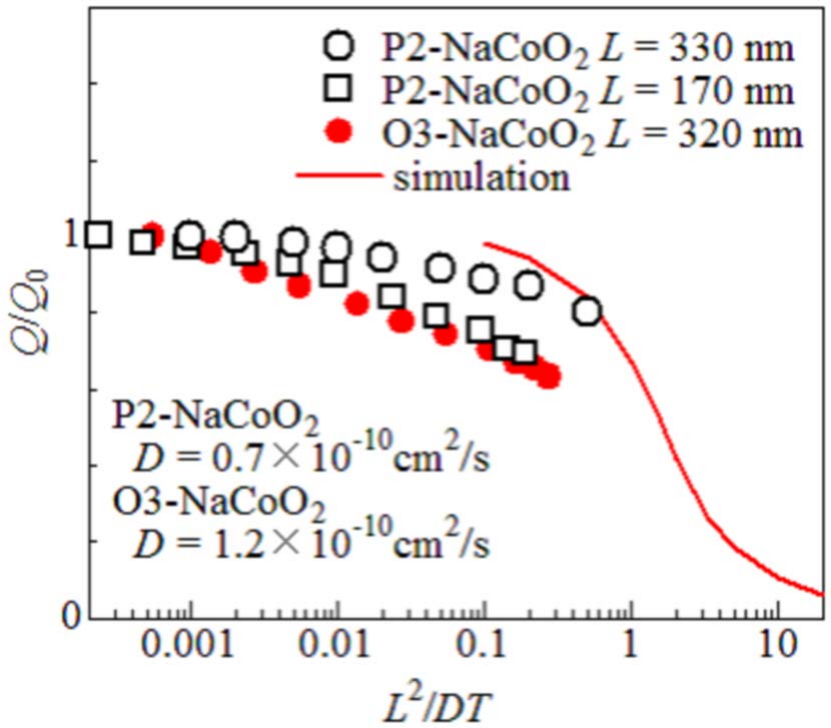
Relative capacity (*Q*/*Q*_0_) against renormalized discharge rate (*L*^2^/*DT*). *L*, *D*, and *T* are the film thickness, Na^+^ diffusion constant, and discharge time, respectively. *Q*_0_ and *Q* are the open-current-voltage (OCV) capacity and actual capacity, respectively. The capacities are evaluated at 2.5 V - *IR*, where *I* and *R* is the current density and battery resistances. Solid curve is the results of simulation based on the one-dimensional diffusion equation. The data of P2-NaCoO_2_ (open squares) were cited from ref. 22.
