# Supplementary material for: Fast discharge process of layered cobalt oxides due to high Na+ diffusion
Source: Sci Rep. 2015 Mar 11;5:9006. doi: 10.1038/srep09006 (PMC4355731; doi:10.1038/srep09006)
Supplement: Supplementary Information [file srep09006-s1.pdf]

Supporting information

Fast discharge process of layered cobalt oxides due to high Na^+ diffusion

Takayuki Shibata[1], Yuya Fukuzumi[2], Wataru Kobayashi[1,2], and Yutaka Moritomo[1,2,3]*

[1]Fucalty of Pure and Applied Science, Univ. of Tsukuba, Tsukuba 305-8571, Japan
[2]College of Physics, Univ. of Tsukuba, Tsukuba 305-8571, Japan
[3]Center for Integrated Research in Fundamental Science and Engineering (CiRfSE), Univ. of Tsukuba, Tsukuba 305-8571, Japan

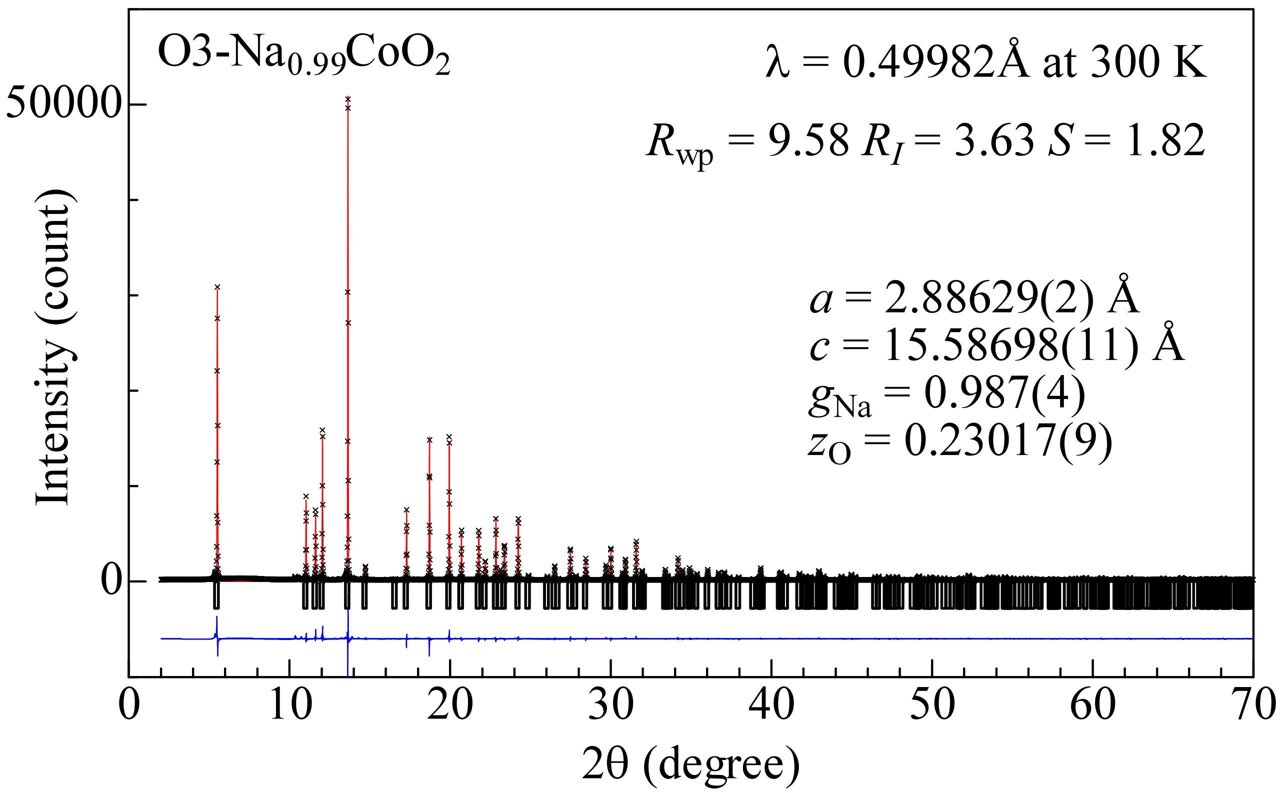

Fig. S1: Rietveld refinement of X-ray powder diffraction pattern of O3-$Na_{0.99}CoO_2$ at 300 K. The synchrotron-radiation X-ray powder diffraction patterns of were measured at BL02B2 beamline of SPring-8. The samples were filled into glass capillaries. The capillaries were sealed and placed on the Debye-Scherrer camera. The powder diffraction patterns were detected with an imaging plate (IP). The exposure time was 5 minutes. The wavelength of the X-ray was calibrated by the lattice constant of standard CeO_2 powders. The diffraction patterns were analyzed by Rietveld method (Rietan-FP) with the hexagonal model ($R\underline{3}m$; Z= 3). Crosses and solid curve are experimental data and Rietveld refinement, respectively. Lower curve is the difference between experiment and calculation. Obtained structural parameters are listed in the following Table.

atom	site	g	x	y	z	B
Na	3a	0.987(4)	0	0	0	0.494(27)
Co	3b	1	0	0	1/2	0.145(8)
O	6c	1	0	0	0.23017(9)	0.269(2)

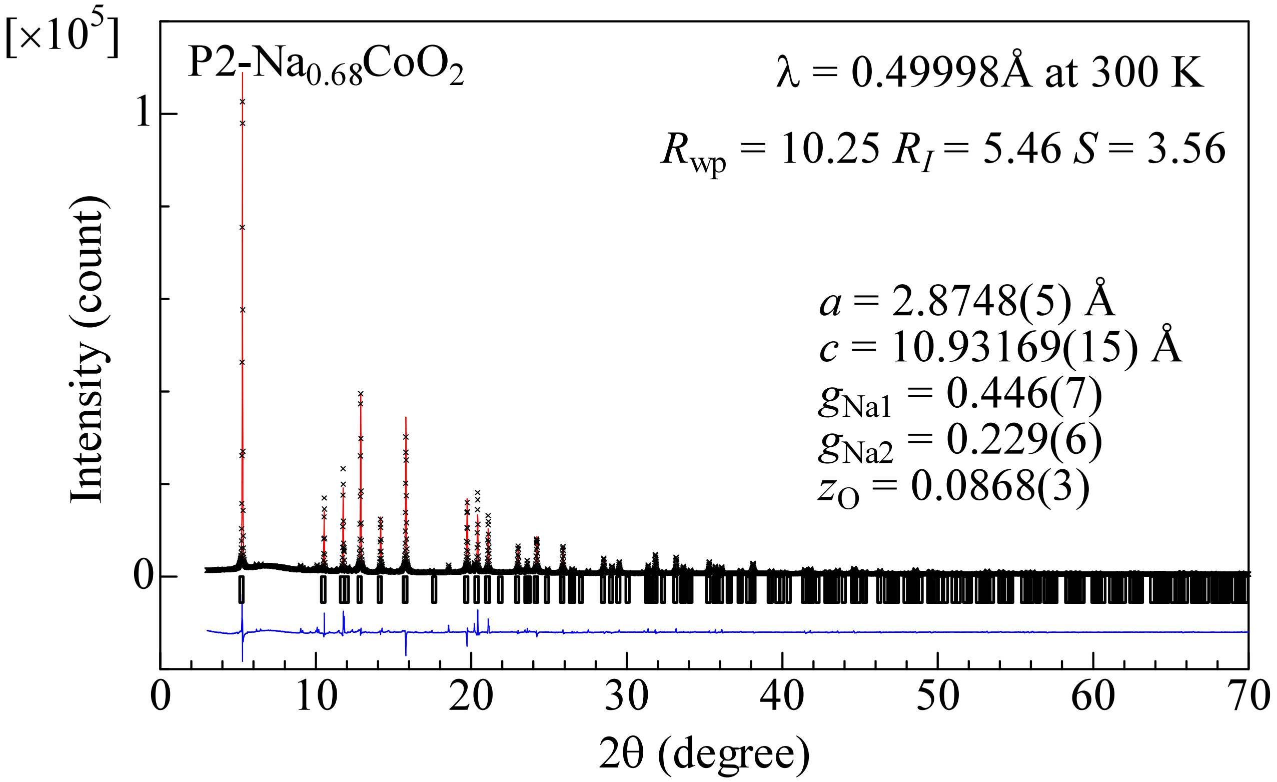

Fig. S2: Rietveld refinement of X-ray powder diffraction pattern of P2-$Na_{0.68}$CoO2 at 300 K. The synchrotron-radiation X-ray powder diffraction patterns of were measured at BL02B2 beamline of SPring-8. The samples were filled into glass capillaries. The capillaries were sealed and placed on the Debye-Scherrer camera. The powder diffraction patterns were detected with an imaging plate (IP). The exposure time was 5 minutes. The wavelength of the X-ray was calibrated by the lattice constant of standard CeO_2. The diffraction patterns were analyzed by Rietveld method (Rietan-FP) with the hexagonal model ($P6_3$/mmc; Z= 2). Crosses and solid curve are experimental data and Rietveld refinement, respectively. Lower curve is difference between experiment and calculation. Obtained structural parameters are listed in the following Table.

atom	site	g	X	y	z	B
Na1	2d	0.446(7)	1/3	2/3	3/4	2.92(24)
Na2	2b	0.229(6)	0	0	1/4	2.92
Co	2a	1	0	0	0	0.23(1)
O	4f	1	1/3	2/3	0.0868(3)	0.57(6)

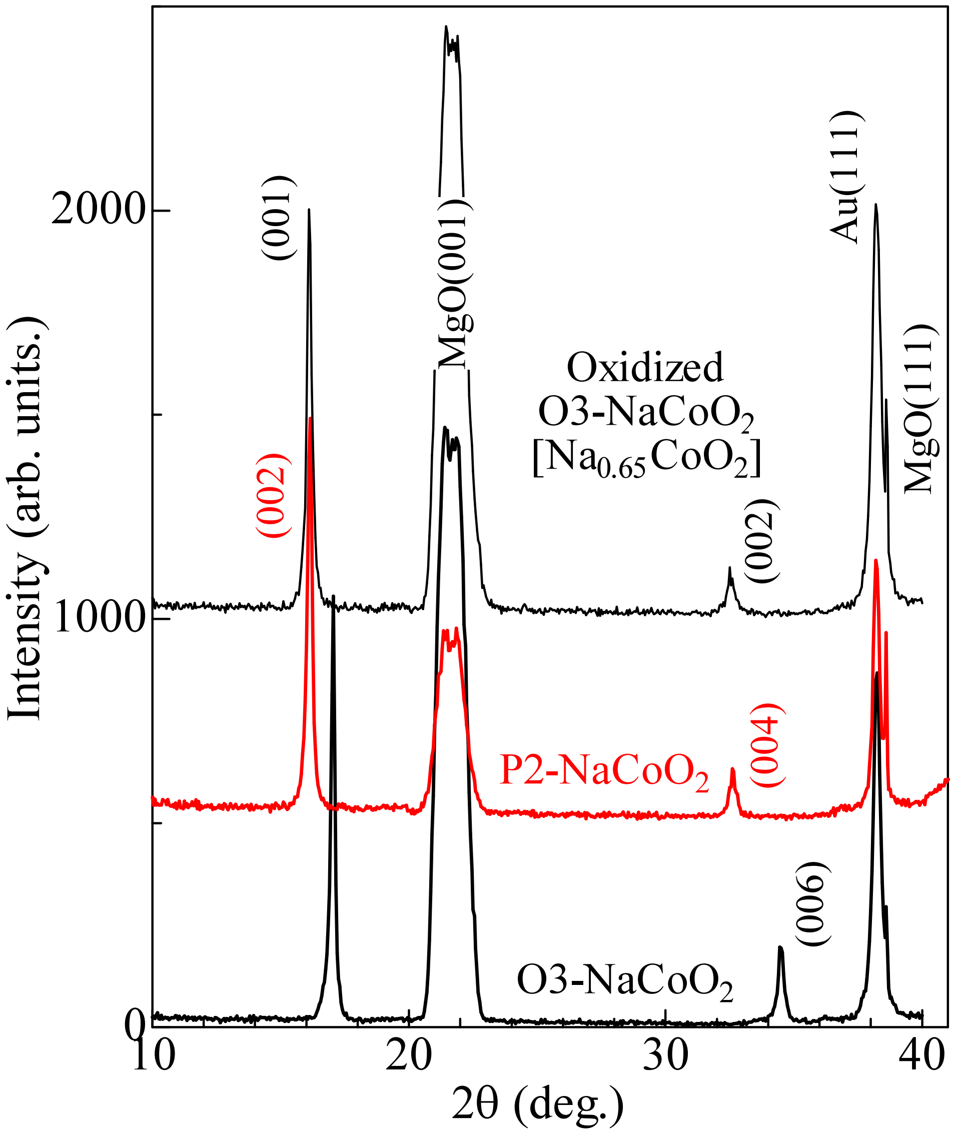

Fig. S3: X-ray diffraction pattern of thin films of O3-$NaCoO_2$, P2-$NaCoO_2$ and oxidized O3-Na_xCoO_2.The reflections of the oxidized O3-$NaCoO_2$ film ($Na_{0.65}CoO_2$) were indexed in a P'3-type monoclinic model ($C2/m$: $Z = 2$). The diffraction patterns were measured in the θ-2θ configuration with the Cu Kα line.

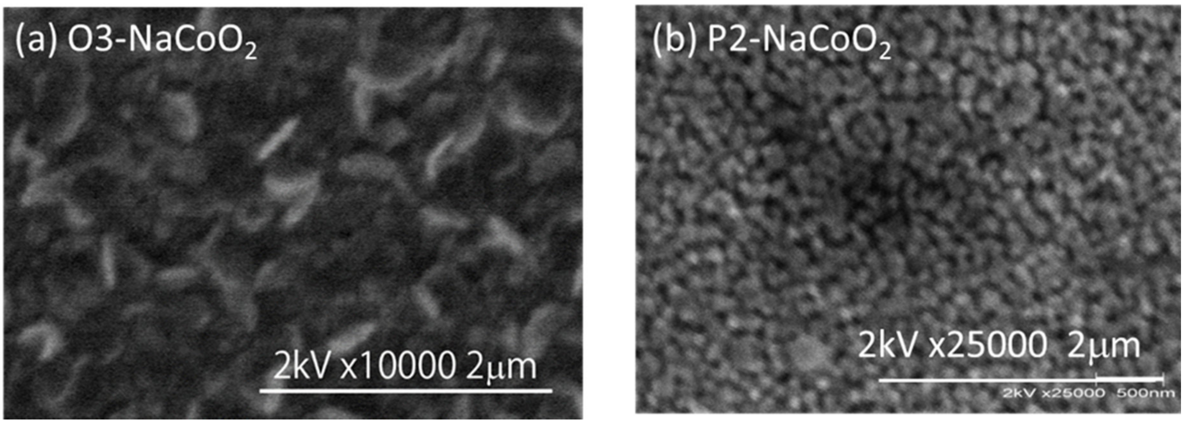

Fig. S4: Surface SEM images of films of (a) O3-$NaCoO_2$ and (b) P2-$NaCoO_2$.

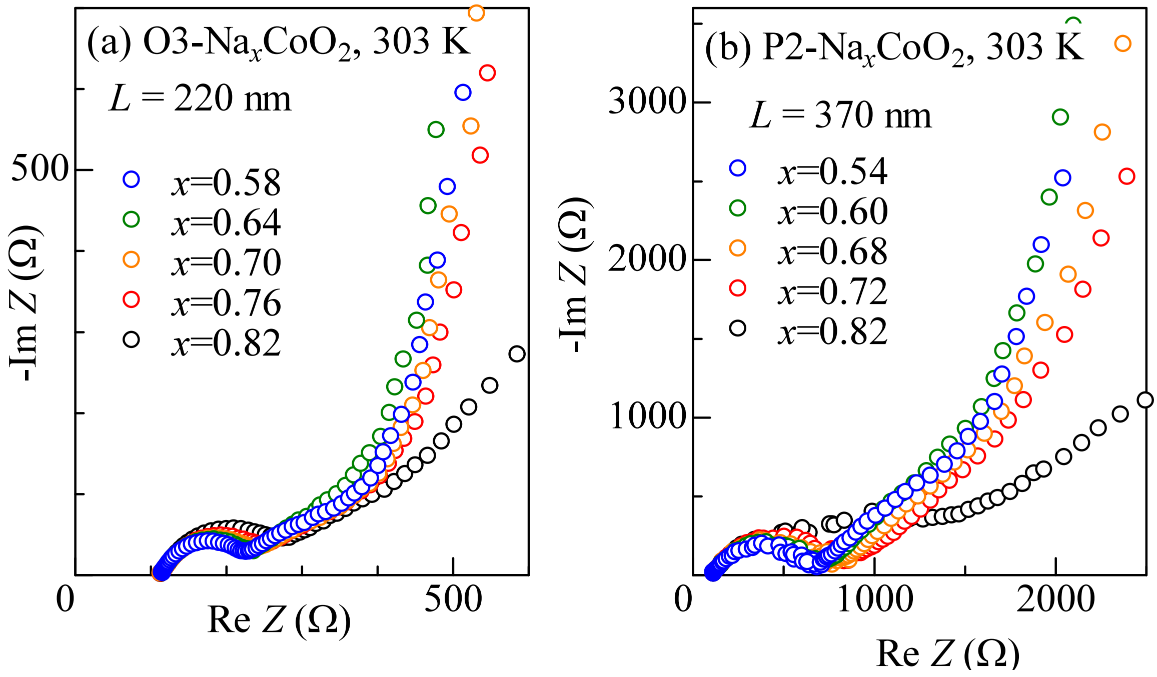

Fig. S5: Complex impedance spectra of films of (a) O3-Na$_x$CoO$_2$ and (b) P2-Na$_x$CoO$_2$ at 303 K against x.

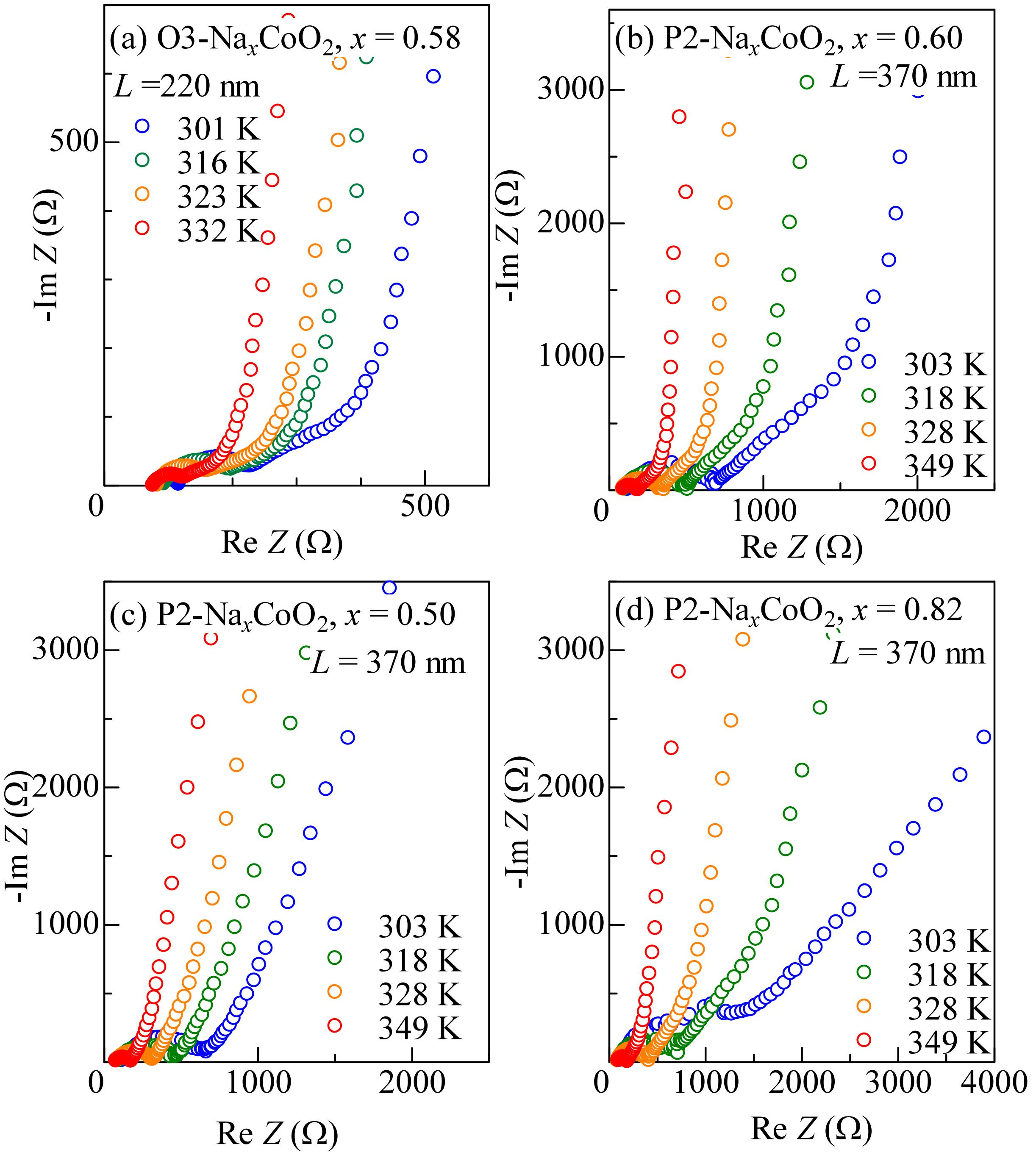

Fig. S6: Complex impedance spectra of films of (a) O3-Na_xCoO_2 and (b) - (c) P2-Na_xCoO_2 against temperatures.

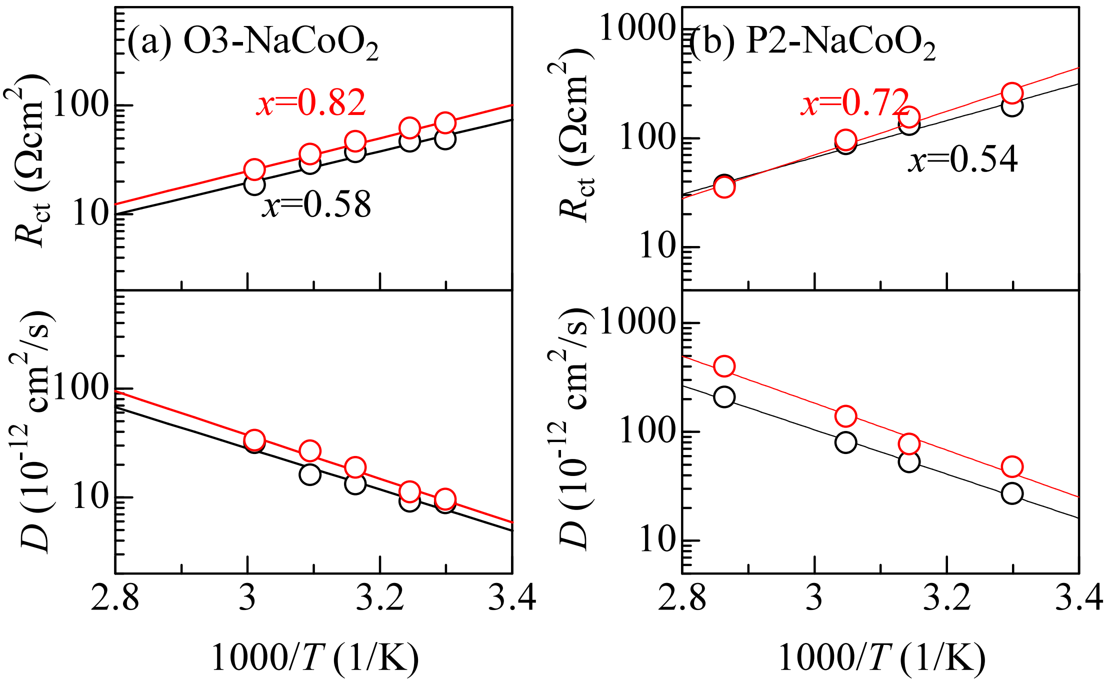

Fig. S7: Arrhenius plot of (a) Na^+ diffusion constant (D) and (b) ionic charge-transfer resistance (R_{ct}) in films of (a) O3-Na_xCoO_2 and (b) P2-Na_xCoO_2.

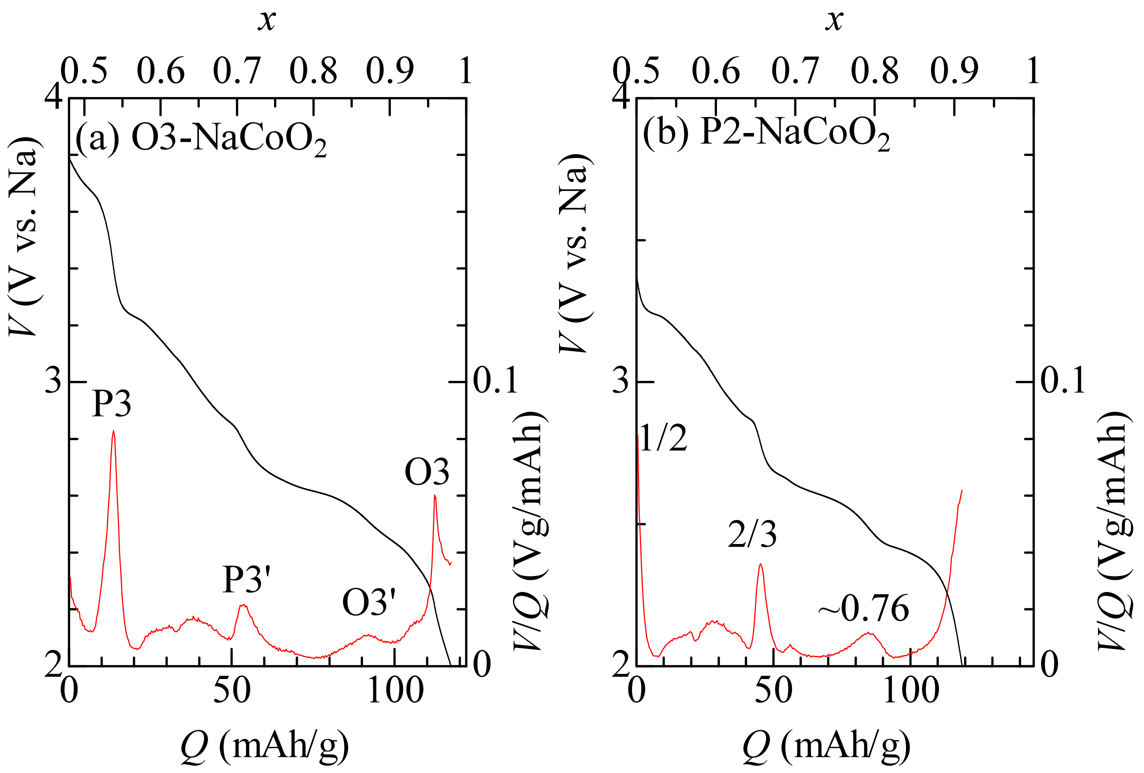

Fig. S8: OCV discharge curves and the plateau structures of (a) O3-$NaCoO_2$ and (b) P2-$NaCoO_2$. Red curve is the *Q*-derivative of *V*. P3, P'3, O'3, O3, 1/2, 2/3, and ~0.76 represent single phases.

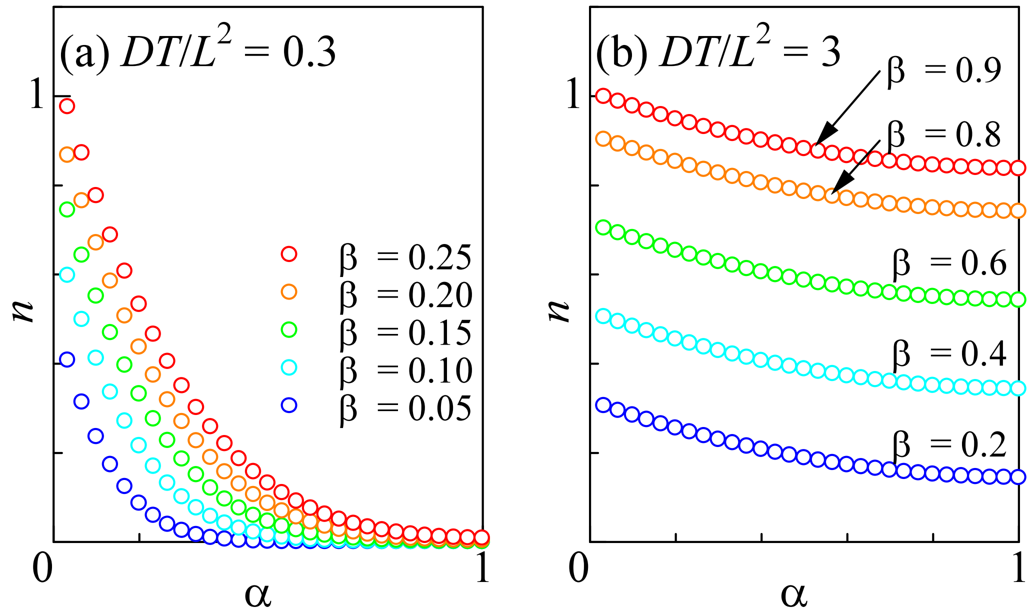

Fig. S9: Numerical calculation of renormalized diffusion equation $\frac{\partial n}{\partial \beta} = \frac{DT}{L^2}\frac{\partial^2 n}{\partial \alpha^2}$, against α. *n, L.* and *T* is the Na^+ density, film thickness, and the time needed for full discharge, respectively. The electrolyte / active material boundary locates at $\alpha = 0$, while active material / collective electrode boundary locates at $\alpha = 1$. The system is fully-discharged at $\beta = 1$. We simulate the discharge process by difference calculus with the space mesh (N_α) of 30 and time mesh (N_β) of 20,000 (Fig. S7). At the active material/electrolyte boundary ($\alpha = 0$), we forces a constant Na^+ intercalation ($=N_\alpha / N_\beta$). The Na^+ intercalation stops if the density ($= 1 - n$) of the Na^+ vacancy at $\alpha = 0$ becomes smaller than N_α / N_β. The β value at this condition corresponds to Q/Q_0.
